# *Notes from the Field:* Fatal *Vibrio anguillarum* Infection in an Immunocompromised Patient — Maine, 2017

**DOI:** 10.15585/mmwr.mm6734a5

**Published:** 2018-08-31

**Authors:** Jennifer A. Sinatra, Kate Colby

**Affiliations:** ^1^Epidemic Intelligence Service, CDC; ^2^Maine Department of Health and Human Services; ^3^University of Southern Maine, Portland, Maine.

In July 2017, a woman aged 65 years was evaluated at a hospital emergency department in Maine for an approximately 10-cm area of necrosis on her left lower leg identified as likely skin and soft tissue infection. The patient noted pain in the area that morning and was unable to walk when examined later that day. Computed tomography indicated extensive cellulitis in the area; she was hospitalized and treated with intravenous antibiotics. The Maine Health and Environmental Testing Laboratory identified *Vibrio anguillarum* from blood cultures collected after admission and before starting treatment; stool and wound cultures were not collected. Approximately 36 hours after she first arrived at the emergency department, the patient developed septic shock and multiorgan failure, dying 2 days after admission.

The patient spent the summer in Maine and lived the rest of the year in a southern coastal state. She was in Maine during her exposure period and illness and had reported vomiting and diarrhea 9 days before seeking treatment at the hospital, which had resolved in 1 day, and one additional diarrhea episode the night before admission. Her husband reported that she had eaten a lobster roll from a local restaurant and a cod loin cooked at home approximately 10 days before the onset of leg pain and a lobster and other seafood dip purchased at a grocery store approximately 5 days before onset of leg pain; he could not recall specifically when each of these foods was consumed in relation to the onset of vomiting and diarrhea. None of the seafood was reported to have been raw or undercooked. The patient had also waded at a beach as recently as the day before seeking treatment, but she did not have any known wounds. Her husband reported that she was bitten by a greenhead fly while at the beach, but the location of the fly bite was not known. A traceback investigation was performed on the seafood she consumed; no informative findings were reported. Her previous medical history included multiple myeloma and amyloidosis, both of which were being treated with immunosuppressive drugs.

Vibriosis is an underrecognized and underreported infection caused by species of the family Vibrionaceae other than toxigenic *Vibrio cholerae* O1 and O139, which cause cholera (*1*,*2*). Typically, Vibrionaceae cause gastrointestinal illness with mild to severe watery diarrhea, although they can also cause bacteremia, wound, or extraintestinal infections. The most common transmission modes involve consumption of raw or undercooked shellfish or contact with seawater. Persons with liver disease, cancer, diabetes, HIV, thalassemia, or who receive immunosuppressive therapy are at increased risk for serious infection. In the United States, vibriosis causes an estimated 80,000 illnesses, 500 hospitalizations, and 100 deaths annually (*2*).

*Vibrio* bacteria are gram-negative bacilli naturally found in coastal waters. Their growth and concentration increases with warmer water temperatures, leading to a seasonal distribution of *Vibrio* infections, with most occurring from summer through early autumn (*3*). *V. anguillarum* (previously known as *Listonella anguillarum*) is normally a pathogen of fish, crustaceans, and bivalves and causes considerable economic losses in the fishing and aquaculture industries (*4*). This is the first reported instance of *V. anguillarum* associated with human illness.

Recent studies report increasing sea surface temperatures in the coastal North Atlantic corresponding with increased abundance and spread of *Vibrio* bacteria and increased *Vibrio*-associated illnesses in the United States (*5*,*6*). Vibriosis is preventable by ensuring that shellfish and seafood are fully cooked before consuming and by either avoiding exposure to seawater when a wound is present or wearing a waterproof dressing and washing wounds after seawater exposure. This immunocompromised patient had exposures to seafood and seawater. With multiple potential exposures, the source and route of infection could not be determined, but two possibilities based on the available epidemiologic data include 1) exposure of a small wound or the fly bite to contaminated seawater resulting in cellulitis and invasive blood infection, or 2) consumption of seafood contaminated with *Vibrio* that caused the diarrhea and the subsequent invasive blood infection and cellulitis. In light of this patient’s comorbidities, determining whether this pathogen was the sole factor in her decline was not feasible, but the case serves as a reminder that laboratories and physicians should be aware of the possibility of illness from *Vibrio* species outside of those commonly observed.
